# Association Between 10-Year Fracture Probability and Nonalcoholic Fatty Liver Disease With or Without Sarcopenia in Korean Men: A Nationwide Population-Based Cross-Sectional Study

**DOI:** 10.3389/fendo.2021.599339

**Published:** 2021-03-31

**Authors:** Hye Jun Lee, Duk Chul Lee, Choon Ok Kim

**Affiliations:** ^1^ Department of Family Medicine, College of Medicine, Yonsei University, Seoul, South Korea; ^2^ Department of Clinical Pharmacology, Severance Hospital, Yonsei University Health System, Seoul, South Korea

**Keywords:** nonalcoholic fatty liver disease, liver fibrosis, sarcopenia, 10-year fracture probability, fracture risk assessment tool

## Abstract

**Objective:**

Nonalcoholic fatty liver disease (NAFLD) and sarcopenia, which are common in elderly men, are known as risk factors of fracture. However, few studies have examined the association with fracture in these patients. Therefore, we aimed to investigate the association between NAFLD with or without sarcopenia and 10-year fracture probability in Korean men aged ≥50 years.

**Materials and Methods:**

Data of 2,525 individuals from the 2010–2011 Korea National Health and Nutrition Examination Survey were analyzed. NAFLD was defined using the fatty liver index (FLI) and comprehensive NAFLD score (CNS), and liver fibrosis using the fibrosis 4 calculator. Sarcopenia was defined as the lowest quintile for sex-specific sarcopenia index cutoff; values. The Fracture Risk Assessment (FRAX) tool was used to predict the 10-year probability of major osteoporotic and hip fractures.

**Results:**

Compared to the no NAFLD group, the 10-year major osteoporotic fracture probability was significantly associated with the FLI-defined (β = 0.16, P = 0.002) and CNS-defined (β = 0.20, P < 0.001) NAFLD groups with liver fibrosis. Similarly, the 10-year hip fracture probability was significantly associated with the FLI- and CNS-defined NAFLD with liver fibrosis groups compared to the group without NAFLD (FLI-defined group, β = 0.04, P = 0.046; CNS-defined group, β = 0.05, P = 0.048). Furthermore, in the group with sarcopenia, the 10-year major osteoporotic fracture probability was significantly associated with the FLI- and CNS-defined NAFLD with liver fibrosis groups compared to the group without NAFLD (FLI-defined group, β = 0.29, P = 0.003; CNS-defined group, β = 0.38, P < 0.001).

**Conclusions:**

NAFLD with liver fibrosis is significantly associated with a higher 10-year major osteoporotic and hip fracture probability in Korean men aged ≥50 years, and this positive association was more profound in patients with sarcopenia. Therefore, screening middle-aged to elderly men who have NAFLD combined with liver fibrosis and sarcopenia may help prevent fractures.

## Introduction

Fractures not only reduce the quality of life, but also increase medical and health care cost, thereby imposing a significant financial and socioeconomic burden (1). Therefore, it is important to predict and prevent fractures. Currently, the Fracture Risk Assessment (FRAX), which is a country-specific instrument developed by the World Health Organization (WHO), is the most widely used fracture prediction tool for individuals aged > 40 years (2). In FRAX, the 10-year probability of both major osteoporotic and hip fractures can be calculated by inputting ten clinical risk factors for fracture (2). Based on FRAX, 37.7% of menopausal women and 12.7% of men aged over 50 years in South Korea are at high risk of osteoporotic fracture (3). Another study also found that South Korean women and men have an average of 59.5% and 23.8% risk of osteoporotic fractures, respectively, in their lifetime (4).

Nonalcoholic fatty liver disease (NAFLD) is one of the most common chronic liver diseases. It initially manifests as mild steatosis or nonalcoholic steatohepatitis, but can progress to liver fibrosis, end-stage liver disease with cirrhosis, and hepatocellular carcinoma (5). The incidence of NAFLD is constantly increasing, and it is associated with liver-related morbidity and mortality (1). NAFLD is considered a hepatic manifestation of metabolic syndrome. It is associated with various metabolic abnormalities and may be associated with an increased risk of fractures (6). However, evidence on the association between NAFLD and bone mineral density (BMD) are conflicting (7). Most studies investigating this association with BMD have targeted mild liver steatosis (8), and few studies have evaluated patients with advanced NAFLD, such as nonalcoholic steatohepatitis (9). Furthermore, there is no direct analysis of the association between NAFLD and fracture using the FRAX tool.

The prevalence of NAFLD is twice as high in men than in women (10), and one study also reported that NAFLD and sarcopenia often co-occur in elderly patients (11). Sarcopenia is associated with decreased bone density and loss of muscle mass, thus making it a risk factor for osteoporotic fracture and a predisposing factor for falls (12). A systematic review also reported that sarcopenia was independently associated with NAFLD and, possibly, advanced fibrosis (13). Therefore, we focused on the effect of NAFLD with liver fibrosis and sarcopenia on the 10-year fracture probability evaluated using the FRAX tool in middle-aged to elderly Korean men. One study that evaluated the 10-year fracture probability using the FRAX tool showed that such probability was increased in middle-aged Korean women with metabolic syndrome (14). However, to the best our knowledge, the 10-year fracture probability evaluated using FRAX tool has not been investigated in patients with NAFLD, liver fibrosis, and sarcopenia. In this study, we hypothesized that NAFLD is significantly associated with a higher 10-year fracture probability, and this association are more profound in patients with sarcopenia. Therefore, we aimed to investigate the association between 10-year fracture risk and NAFLD with or without sarcopenia in Korean men aged ≥50 years, using data from the Korea National Health and Nutrition Examination Survey (KNHANES).

## Materials and Methods

### Study Population and Design

This cross-sectional study used data from the 2010–2011 KNHANES. The KNHANES is a nationwide population-based survey conducted by the Korean Ministry of Health and Welfare and the Division of Chronic Disease Surveillance of the Korean Centers for Disease Control and Prevention.

**Figure 1:**
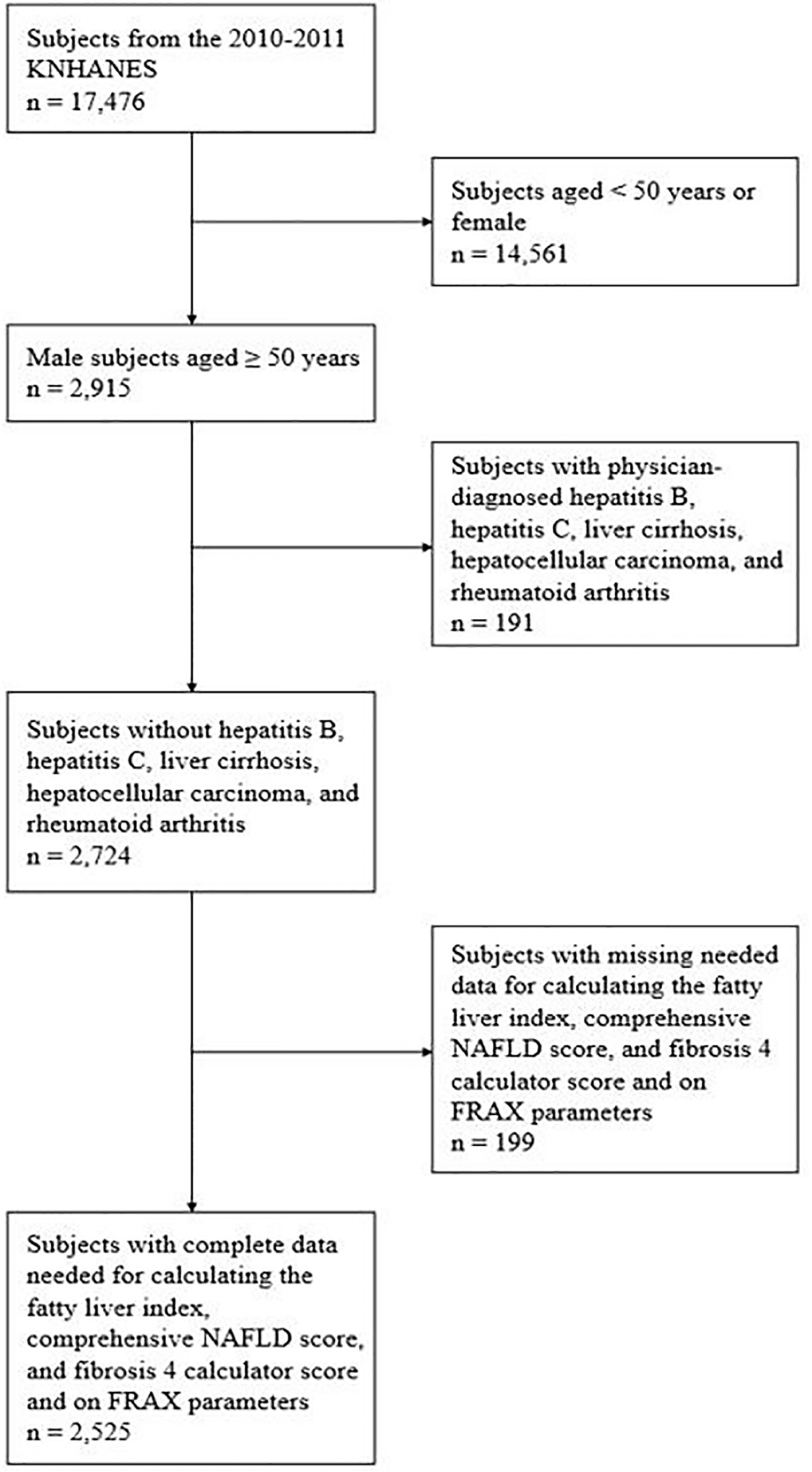
Subject inclusion flow chart. KNHANES, Korea National Health and Nutrition Examination Survey; NAFLD, nonalcoholic fatty liver disease; FRAX, fracture risk assessment.

Among the 17,476 subjects of the 2010–2011 KNHANES, 2,915 men aged ≥50 years were initially evaluated. Of them, we excluded 191 patients with physician-diagnosed hepatitis B, hepatitis C, liver cirrhosis, hepatocellular carcinoma, and rheumatoid arthritis, which is risk factor for fracture and patients who were high-risk drinkers (> 210 g per week for men and > 140 g per week for women). From the remaining 2,724 patients, those with missing data on factors needed for calculating the fatty liver index (FLI), comprehensive NAFLD score (CNS), fibrosis 4 calculator (FIB-4), and FRAX were further excluded. Finally, 2,525 subjects were included in the final analysis (**Figure 1**).

All participants provided written informed consent before participation in the study, and the KNHANES was conducted following ethical approval by the Institutional Review Board of the Korea Centers for Disease Control and Prevention (No. 2010-02CON-21-C, 2011-02CON-06C). The protocol of the current study was approved by the Institutional Review Board of Yonsei University, Seoul, Korea (No. 4-2020-0744).

### Data Collection

Subjects were interviewed by trained staff using standardized health questionnaires, which included questions on demographic information, medical history of osteoporosis diagnosis or fracture, parental history of fracture, history of smoking, and alcohol intake. In our study, the variables used in the FRAX tool to predict the 10-year probability of major osteoporotic fracture (spine, forearm, hip, or shoulder fractures) and hip fracture (2) were considered when adjusting for the risk factors. The variables included age, sex, previous history of fracture, parental history of hip fracture, prevalence of rheumatoid arthritis, secondary osteoporosis, use of steroids, current smoking, drinking more than 3 units per day, body weight, height, and bone mineral density of the femoral neck (2). Anthropometric measurements and laboratory tests were also conducted.

NAFLD was defined using FLI and CNS, and liver fibrosis using FIB-4. The FLI model was calculated using four variables: body mass index (BMI), waist circumference, serum triglycerides (TG), and serum γ-glutamyl transferase (15). The CNS model was calculated using the parameters of age, BMI, waist circumference, prevalence of diabetes, alcohol intake, exercise, serum fasting glucose, serum TG, serum high density lipoprotein-cholesterol, serum uric acid, serum aspartate aminotransferase, and serum alanine aminotransferase (16). A FLI of ≥60 and a CNS of ≥40 were considered indicative of NAFLD (15, 16). The FIB-4 score was calculated using the parameters of age, plasma platelet count, serum aspartate aminotransferase, and serum alanine aminotransferase, with liver fibrosis defined as scores above 1.3 (17). According to the presence of NAFLD and liver fibrosis, it was classified into three groups; no NAFLD group, NAFLD without liver fibrosis group, and NAFLD with liver fibrosis group.

Sarcopenia was determined using the sarcopenia index (sarcopenia index = total appendicular skeletal muscle mass (kg)/BMI (kg/m^2^)), which involved measurement of the appendicular skeletal muscle mass *via* dual-energy x-ray absorptiometry (DEXA, QDR 4500A; Hologic, Bedford, MA). Sarcopenia was defined as the lowest quintile for sex-specific sarcopenia index cutoff values (< 0.807 for men) based on a modified recommendation from the Foundation for the National Institutes of Health (18). BMD of the femoral neck was also examined using DEXA. BMI was calculated by dividing weight (kg) with the square of height (m^2^). Smoking was defined as current smoking. Monthly alcohol intake was defined as drinking more than once a month during the past year.

### Statistical Analysis

The characteristics of the subjects classified according to the presence of NAFLD and liver fibrosis were expressed as the means and standard deviations for continuous variables and as numbers and percentages for categorical variables. A one-way analysis of variance was used to compare the continuous variables and a chi-squared test for categorical variables. For *post hoc* analysis, the Bonferroni method was used.

Multivariate linear regression analysis was performed to analyze the association between NAFLD with liver fibrosis and sarcopenia and the 10-year major osteoporotic and hip fracture probability. The regression coefficients (β) and 95% confidence intervals (CIs) were estimated. We adjusted for multiple variables that showed significant associations in the univariate analysis and those based on clinical relevance. After calculating the crude β (model 1), model 2 was adjusted for age, osteoporosis diagnosis, fracture history, parental history of fracture, BMI, BMD of the femoral neck, current smoking, and alcohol intake. All variables in the linear regression analysis were examined for multi-collinearity, and only those variables with a variance inflation factor of < 5 were used. All statistical analyses were performed using IBM SPSS version 25 (Armonk, NY, USA). All *P* values were two-tailed, and a *P* value of < 0.05 was considered statistically significant.

## Results

### Subject Characteristics

A total of 2,525 subjects were included in this study (**Figure 1**). **Table 1** shows the demographic characteristics of the subjects according to NAFLD and liver fibrosis. The prevalence rates of FLI- and CNS-defined NAFLD were 20% (n = 512) and 46% (n = 1158), respectively. The prevalence rate for NAFLD accompanied by FIB-4–defined liver fibrosis was 11% (n = 279) in patients with FLI-defined NAFLD and 24% (n = 614) in patients with CNS-defined NAFLD.

**Table 1:** Demographic characteristics of the study population.

	FLI-defined NAFLD			*P* value	CNS-defined NAFLD			*P* value
	NAFLD (-)Fibrosis (-)	NAFLD (+)Fibrosis (-)	NAFLD (+)Fibrosis (+)		NAFLD (-)Fibrosis (-)	NAFLD (+)Fibrosis (-)	NAFLD (+)Fibrosis (+)	
	(n = 2013)	(n = 233)	(n = 279)		(n = 1367)	(n = 544)	(n = 614)	
Age (years)	64.1 ± 8.6	57.35 ± 6.26^a^	63.71 ± 7.51^b^	< 0.001	64.30 ± 8.83	58.67 ± 6.92^a^	65.63 ± 7.58^a,b^	< 0.001
BMI (kg/m^2^)	22.99 ± 2.53	26.54 ± 2.30^a^	26.35 ± 2.62^a^	< 0.001	22.02 ± 2.23	25.61 ± 2.18^a^	25.71 ± 2.27^a^	< 0.001
BMD, femoral neck (g/cm^2^)	0.74 ± 0.12	0.79 ± 0.11^a^	0.76 ± 0.11^a^	< 0.001	0.72 ± 0.12	0.79 ± 0.11^a^	0.76 ± 0.11^a,b^	< 0.001
Diagnosis								
Osteoporosis (n, %)	27 (2.0)	3 (2.0)	2 (1.1)	0.715	23 (2.5)	5 (1.4)	4 (1.0)	0.123
Sarcopenia (n, %)	284 (21.3)	51 (32.9)^a^	78 (43.1)^a^	< 0.001	163 (18.3)	99 (26.6)^a^	151 (37.4)^a,b^	< 0.001
History								
Fracture (n, %)	198 (14.9)	22 (14.6)	23 (13.0)	0.795	132 (14.7)	50 (14.0)	61 (15.3)^b^	0.890
Parental fracture (n, %)	154 (11.4)	24 (15.6)	18 (9.9)	0.227	104 (11.4)	55 (14.9)	37 (9.0)	0.039
Smoking (n, %)	626 (31.1)	101 (43.3)^a^	97 (34.8)	0.001	472 (34.5)	191 (35.1)	161 (26.2)^a,b^	< 0.001
Alcohol (n, %)	1315 (65.3)	197 (84.5)^a^	233 (83.5)^a^	< 0.001	864 (63.2)	419 (77.0)^a^	462 (75.2)^a^	< 0.001
10-year probability								
Major fracture (%)	1.67 ± 1.49	1.94 ± 1.80^a^	1.75 ± 1.66	0.042	1.60 ± 1.47	1.92 ± 1.60^a^	1.76 ± 1.63	< 0.001
Hip fracture (%)	0.18 ± 0.31	0.11 ± 0.12^a^	0.19 ± 0.32^b^	0.001	0.18 ± 0.31	0.12 ± 0.13^a^	0.22 ± 0.36^a,b^	< 0.001

Data were obtained from the 2010–2011 Korean National Health and Nutrition Examination Survey.

P values were calculated using a one-way analysis of variance or chi-squared test. The Bonferroni method was used for post hoc analysis.

Continuous variables are expressed as the means and standard deviations, whereas categorical variables are expressed as numbers and percentages.

a P <0.05 by post hoc analyses when compared without NAFLD and liver fibrosis.

b P <0.05 by post hoc analyses when compared without liver fibrosis but with NAFLD.

NAFLD, nonalcoholic fatty liver disease; FLI, fatty liver index; CNS, comprehensive NAFLD score; BMI, body mass index; BMD, bone mineral density.

In the analysis of the three groups of NAFLD defined by FLI and CNS, age was the youngest and the 10-year hip fracture probability was the lowest in the NAFLD without liver fibrosis group (P < 0.001 and P = 0.001, respectively). Meanwhile, the BMI, BMD of the femoral neck, prevalence of sarcopenia, and alcohol intake were the lowest in the group without NAFLD (all P < 0.001). The 10-year major osteoporotic fracture probability was also the lowest in the group without NAFLD (P = 0.042 for FLI-defined NAFLD; P < 0.001 for CNS-defined NAFLD).

### Association Between NAFLD With FIB-4 Defined Liver Fibrosis and 10-Year Major Osteoporotic and Hip Fracture Probability

**Table 2** shows the β and 95% CIs for the 10-year major osteoporotic fracture probability according to FLI- and CNS-defined NAFLD with FIB-4–defined liver fibrosis in the overall cohorts. The model was adjusted for age, osteoporosis diagnosis, previous and parental history of fracture, BMD of the femoral neck, BMI, current smoking, and alcohol intake.

**Table 2:** Association between NAFLD with FIB-4–defined liver fibrosis and 10-year major osteoporotic fracture probability in Korean men aged ≥50 years.

	FLI-defined NAFLD			CNS-defined NAFLD		
	NAFLD (-) Fibrosis (-)	NAFLD (+) Fibrosis (-)	NAFLD (+) Fibrosis (+)	NAFLD (-) Fibrosis (-)	NAFLD (+)Fibrosis (-)	NAFLD (+) Fibrosis (+)
Model 1						
β	Reference	0.28*	0.06	reference	0.19*	0.06
95% CI	Reference	0.06–0.50	-0.15–0.27	reference	0.01–0.37	-0.11–0.24
*P* value	Reference	0.013	0.588	reference	0.040	0.481
Model 2						
β	Reference	0.07	0.16*	reference	0.05	0.20*
95% CI	Reference	-0.03–0.18	0.06–0.25	reference	-0.04–0.14	0.10–0.29
*P* value	Reference	0.150	0.002	reference	0.245	< 0.001

Model 1 was the crude model.

Model 2 was adjusted for age, osteoporosis diagnosis, fracture history, parental history of fracture, body mass index, bone mineral density of the femoral neck, current smoking, and alcohol intake.

β is the regression coefficient, and statistical significance is represented by 95% the confidence intervals and P values.

NAFLD, nonalcoholic fatty liver disease; FIB-4, fibrosis 4 calculator; FLI, fatty liver index; CNS, comprehensive NAFLD score.

*indicates statistical significance at the P value <0.05 level.

The 10-year major osteoporotic fracture probability was higher in the FLI- and CNS-defined NAFLD without liver fibrosis groups than that in the group without NAFLD, although the difference was not significant. However, compared to the no NAFLD group, the 10-year major osteoporotic fracture probability showed significant positive associations with the FLI-defined (β = 0.16, P = 0.002) and CNS-defined (β = 0.20, P < 0.001) NAFLD groups with liver fibrosis.

For the 10-year hip fracture probability, compared with the group without NAFLD, the FLI- and CNS-defined NAFLD with liver fibrosis groups showed significant positive associations between the fracture probability and NAFLD with liver fibrosis (**Table 3**, FLI-defined group, β = 0.04, P = 0.046; CNS-defined group, β = 0.05, P = 0.048).

**Table 3:** Association of NAFLD with FIB-4–defined liver fibrosis and 10-year hip fracture probability in Korean men aged ≥50 years.

	NAFLD by FLI			NAFLD by CNS		
	NAFLD (-) Fibrosis (-)	NAFLD (+) Fibrosis (-)	NAFLD (+)Fibrosis (+)	NAFLD (-) Fibrosis (-)	NAFLD (+) Fibrosis (-)	NAFLD (+) Fibrosis (+)
Model 1						
β	Reference	-0.03	0.10 *	Reference	-0.03	0.13 *
95% CI	Reference	-0.07–0.02	0.06–0.14	Reference	-0.07–0.01	0.09–0.18
*P* value	Reference	0.210	< 0.001	Reference	0.170	< 0.001
Model 2						
β	Reference	-0.01	0.04 *	Reference	-0.01	0.05 *
95% CI	Reference	-0.05–0.03	0.001–0.08	Reference	-0.05–0.03	0.001–0.09
*P* value	Reference	0.613	0.046	Reference	0.622	0.048

Model 1 was the crude model.

Model 2 was adjusted for age, osteoporosis diagnosis, fracture history, parental history of fracture, body mass index, bone mineral density of the femoral neck, current smoking, and alcohol intake.

β is the regression coefficient, and statistical significance is represented by the 95% confidence intervals and P values.

NAFLD, nonalcoholic fatty liver disease; FIB-4, fibrosis 4 calculator; FLI, fatty liver index; CNS, comprehensive NAFLD score

*indicates statistical significance at the P value <0.05 level.

### Association Between NAFLD With Significant Liver Fibrosis and 10-Year Major Osteoporotic Fracture Probability in the Patients With Sarcopenia

**Table 4** shows the β and 95% CIs for the 10-year major osteoporotic fracture probability according to FLI- and CNS-defined NAFLD with FIB-defined liver fibrosis in 413 men with sarcopenia aged ≥50 years. This model was also adjusted for age, osteoporosis diagnosis, previous or parental history of fracture, BMD of the femoral neck, BMI, current smoking, and alcohol intake.

**Table 4:** Association of NAFLD with FIB-4 defined liver fibrosis and 10-year major osteoporotic fracture probability in Korean men with sarcopenia aged ≥50 years.

	NAFLD by FLI			NAFLD by CNS		
	NAFLD (-)Fibrosis (-)	NAFLD (+)Fibrosis (-)	NAFLD (+)Fibrosis (+)	NAFLD (-)Fibrosis (-)	NAFLD (+)Fibrosis (-)	NAFLD (+)Fibrosis (+)
Model 1						
β	Reference	0.20	0.03	Reference	0.19	0.05
95% CI	Reference	-0.20–0.59	-0.32–0.38	Reference	-0.19–0.57	-0.31–0.40
*P* value	Reference	0.322	0.848	Reference	0.319	0.806
Model 2						
β	Reference	0.09	0.29 *	Reference	0.16	0.38 *
95% CI	Reference	-0.11–0.29	0.10–0.47	Reference	-0.04–0.36	0.18–0.58
*P* value	Reference	0.361	0.003	Reference	0.108	< 0.001

Model 1 was the crude model.

Model 2 was adjusted for age, osteoporosis diagnosis, fracture history, parental history of fracture, body mass index, bone mineral density of the femoral neck, current smoking, and alcohol intake.

β is the regression coefficient, and statistical significance is represented by 95% confidence intervals and P values.

NAFLD, nonalcoholic fatty liver disease; FIB-4, fibrosis 4 calculator; FLI, fatty liver index; CNS, comprehensive NAFLD score.

*indicates statistical significance at the P value <0.05 level.

Compared with the group without NAFLD, the 10-year major osteoporotic fracture probability was significantly higher in the FLI-defined (β = 0.29, P = 0.003) and CNS-defined (β = 0.38, P < 0.001) NAFLD with liver fibrosis groups.

## Discussion

This cross-sectional study of data from the 2010–2011 KNHANES showed that NAFLD with liver fibrosis and sarcopenia were significantly associated with the 10-year major osteoporotic probability in Korean men aged ≥50 years. Therefore, we suggested that developing preventive and pharmacological approaches for the management of NAFLD with liver fibrosis and sarcopenia are needed as a preventive strategy for fracture (6).

Known risk factors for osteoporosis and fracture, such as poor nutrition, excess alcohol intake, hypogonadism, and corticosteroid use, are commonly found in patients with chronic liver disease (8). In addition, chronic liver disease in adults (e.g., cirrhosis and advanced liver disease) is a risk factor itself for osteoporosis and increased bone fragility (19). Several studies reported a 10-56% reduction in BMD in adult patients with liver fibrosis or viral liver cirrhosis (20, 21). These results suggest that advanced liver fibrosis, which has progressed in the absence of hepatic decompensation, has a negative effect on bone mass and quality (22). However, there is no research on the direct association between NAFLD with liver fibrosis and fracture probability. Further, the pathophysiological mechanisms by which NAFLD is associated with fractures have not been well established (6).

Our results showed a higher risk of fracture in the group with NAFLD with liver fibrosis, which could be explained by the following mechanisms. Insulin-like growth factor-1 is an important driver of bone formation from mesenchymal stem cells and promotes osteoblast survival and differentiation (23). It is produced primarily in the liver and can lead to impaired production during liver disease (24), likely resulting in a lower bone turnover (25). In addition, excess fat accumulation in the liver causes low-grade, chronic inflammation associated with the development of bone loss and osteoporosis and a higher likelihood of fractures (26). Increased osteoclast activity is mediated by osteoclastogenic proinflammatory cytokines. For example, interleukin 1 and tumor necrosis factor (TNF) are involved in hepatic inflammation and fibrosis (8). In particular, TNF-α can reduce trabecular and cortical bone formation by inhibiting osteoblast differentiation and collagen secretion and by inducing osteoblast apoptosis (27). This cytokine can also promote trabecular and cortical bone resorption by inducing the expression of the receptor activator of nuclear factor kappa B ligand, which inhibits osteoclast activation and osteoblast apoptosis (28).

Sarcopenia increases the risk of falls and fractures by reducing mobile function and increasing frailty and imbalance (10). A systematic review reported a high prevalence of sarcopenia in men with fragility fractures and that sarcopenia was a risk factor for fractures when accompanied by low BMD in men (29). In our study, the association of NAFLD with liver fibrosis and 10-year major osteoporotic fracture probability increased in the group with sarcopenia, indicating the synergic effect of NAFLD with liver fibrosis and sarcopenia on fracture. Although the exact mechanism is unknown, one study of elderly patients reported that NAFLD and sarcopenia share common pathophysiological mechanisms that may increase the risk of fracture, including insulin resistance and chronic low-grade inflammation (13).

Our study had some limitations. First, because this was a cross-sectional study, causal inference cannot be drawn. Further larger longitudinal prospective studies with multiple comparative groups are needed to establish precise interrelationships. Second, among the variables included in the FRAX tool (2), the history of steroid uses and diseases that could cause secondary osteoporosis was not included in the analysis. Therefore, the fracture risk may have been underestimated. In addition, there are other risk factors of fracture (e.g., physical activity, calcium/vitamin D intake) that are not included in the FRAX tool. Therefore, further research including additional confounding factors is needed (30). Third, the gold standard for NAFLD diagnosis is liver biopsy. However, NAFLD and liver fibrosis were diagnosed in our study using FLI, CNS, and FIB-4. It should be noted, however, that liver biopsy is not routinely used in epidemiological studies or actual practice due to its invasiveness and potential for complication (31). In addition, the FLI has relatively high accuracy for Koreans, with an area under receiver operating characteristic curve (AUROC) of 0.86 (16). The CNS is a prediction model developed using data from 15,676 Koreans diagnosed *via* ultrasound. Further, its validation in 66,868 independent examination cohorts yielded a relatively high AUROC value of 0.8-0.9 (16). FIB-4 has also been validated to be a relatively accurate method for diagnosing NAFLD in various races (17). Therefore, the above three diagnostic tools can be considered reliable and easily accessible for the diagnosis of NAFLD and liver fibrosis.

Despite these limitations, this study is valuable because to our best knowledge, this is the first to investigate the association between NAFLD with liver fibrosis and sarcopenia and the 10-year fracture probability. It also provides evidence on the potential of NAFLD with more advanced liver fibrosis and sarcopenia as a therapeutic target for reducing the risk of fractures. The potential impact and precise pathophysiological mechanisms by which NAFLD with liver fibrosis and sarcopenia is associated with the development of fracture warrants further study (32).

In conclusion, this quantitative cross-sectional analysis of data from the 2010–2011 KNHANES revealed that the 10-year major osteoporotic and hip fracture probability was significantly associated with FLI- and CNS-defined NAFLD with liver fibrosis. Notably, this association was more profound in the subjects with sarcopenia. These findings indicate that screening middle-aged to elderly men who have NAFLD combined with liver fibrosis and sarcopenia for the prediction of further fracture may be a potential preventive strategy against future fractures (32).

## Data Availability Statement

The original contributions presented in the study are included in the article/supplementary material. Further inquiries can be directed to the corresponding authors.

## Ethics Statement

All participants provided written informed consent before participation in the study, and the KNHANES was conducted following ethical approval by the Institutional Review Board of the Korea Centers for Disease Control and Prevention (No. 2010-02CON-21-C, 2011-02CON-06C). This study was performed in line with the principles of the Declaration of Helsinki. Approval was granted by the Institutional Review Board of Yonsei University, Seoul, Korea (Approval No. 4-2020-0744).

## Author Contributions

HL, DL, and CK: contributed to the study design. HL: performed data collection, interpretation, and analysis. HL and CK participated in the writing and modification of the article. DL and CK: performed critical review. All authors contributed to the article and approved the submitted version.

## Conflict of Interest

The authors declare that the research was conducted in the absence of any commercial or financial relationships that could be constructed as a potential conflict of interest.
